# Evaluation of Physical Health in an In-Patient Psychiatric Rehabilitation Setting

**DOI:** 10.1192/bjo.2022.184

**Published:** 2022-06-20

**Authors:** Alastair Cockburn, Andrew Watson, Debbie Mountain, Stephen Lawrie

**Affiliations:** 1 Royal Edinburgh Hospital, Edinburgh, United Kingdom; 2 Oxford University Hospitals Trust, Oxford, United Kingdom; 3 Edinburgh University, Edinburgh, United Kingdom

## Abstract

**Aims:**

The mortality gap between patients with serious mental illness (SMI) and without is around 15–20 years. This has multiple contributing factors including poor physical health, side effects of antipsychotic medications and sub-optimal medical management. Presented here is a detailed cross-sectional study of physical health measures in an in-patient rehabilitation population in Scotland. Results are compared to national averages and clinical guidelines with the aims to a) benchmark physical health in this population and b) where possible improve physical health.

**Methods:**

Physical health data including observations, blood tests, and investigations was collected ahead of detailed structured interviews and physical exams performed by a post-foundation doctor. These results were compared to recommendations for physical health monitoring from numerous national and government guidelines (SIGN, NICE, Scottish Government, Maudsley). Data were collected in 4 domains, 1) Indicators of physical health, 2) Engagement with physical health, 3) Concordance with guidelines and 4) Outcomes of reviews.

**Results:**

Data were collected from 57 of all 62 in-patients. 34 reported being generally happy with their health vs 15 unhappy. 42% were obese (compared to 28% of the general population), 84% were smokers (vs 16% in the local population) 16% were hypertensive, 22% had raised HbA1c, 50% had raised cholesterol, 47% had QRISK >10%. 68% agreed to a full physical health review, 65% agreed to flu vaccination. Completed cancer screening uptake compared to the Scottish population was low; Cervical (30% vs 71%), Bowel (8% vs 59%), Breast (23% vs 72%), AAA (0% vs 84%). Patients were generally up to date in terms of recorded weight (100%), BP (98.2%), HR (98.2%) and lipids (89.4%), but not ECG's (61.4%) and Diabetes screening (59.6%). 17 referrals were made to medics/surgeons, 29 to MDT's, 24 medications started, 9 stopped and 27 changed, most commonly statins (12 patients), vitamin D (8 patients) and hypoglycemics (5 patients).

**Conclusion:**

Cardiovascular disease indicators were notably raised, uptake of screening was very poor and there were areas where the service didn't meet national guidelines. The number of referrals and medication changes suggest an unmet need within such services. The findings, if generalisable across similar populations, suggest that more can be done to address ongoing poor physical health in populations with SMI and indeed patient readiness to comply with physical health screening. Screening for key physical health parameters needs to be augmented by working to engage patients and following up with management plans for abnormalities found.

